# Distal cholangiocarcinoma in a patient with portal annular pancreas and aberrant common hepatic artery: a case report

**DOI:** 10.3389/fsurg.2026.1895465

**Published:** 2026-09-28

**Authors:** Xinglong Wang, Weikang Liu, Jirong Shi, Xiaodong Tian

**Affiliations:** Department of Hepatobiliary and Pancreatic Surgery, Peking University First Hospital, Beijing, China

**Keywords:** aberrant hepatic artery, anatomical variation, distal cholangiocarcinoma, pancreaticoduodenectomy, portal annular pancreas

## Abstract

**Background:**

Portal annular pancreas (PAP) is a rare congenital anatomical variation characterized by pancreatic parenchyma encircling the portal vein (PV) or superior mesenteric vein (SMV). An aberrant common hepatic artery (CHA) originating from the superior mesenteric artery (SMA) represents another uncommon vascular anomaly. The coexistence of these two variations with a distal common bile duct (CBD) malignancy is extremely rare and poses significant technical challenges for pancreaticoduodenectomy (PD).

**Case presentation:**

We report a case of a 55-year-old male with obstructive jaundice caused by distal cholangiocarcinoma, concurrent with PAP (Joseph Type IIIb) and a CHA arising from the SMA (Michels type IX). Preoperative imaging, including multiphasic contrast-enhanced computed tomography (CT) with arterial and portal venous phase protocols and three-dimensional vascular reconstruction, meticulously delineated the complex anatomy—including the complete course of the aberrant CHA, the origin of the gastroduodenal artery (GDA), the relationship of the CHA to the pancreas, PV, and CBD, and the main pancreatic duct (MPD) coursing anterior to the PV. A classical pancreaticoduodenectomy (Whipple procedure) was successfully performed. Key surgical adaptations included a phased pancreatic transection strategy to address the annular pancreas—creating two transection surfaces with *en bloc* resection of the posterior pancreatic tissue—and careful dissection to preserve the aberrant hepatic artery. The patient recovered uneventfully with only a biochemical leak and without clinically relevant postoperative pancreatic fistula (POPF, Grade B/C), delayed gastric emptying, post-pancreatectomy hemorrhage, or bile leakage.

**Conclusion:**

This case underscores the critical importance of detailed preoperative radiological evaluation in identifying complex anatomical variations. Individualized surgical planning, guided by precise anatomical understanding, is paramount for safe and successful PD in such challenging scenarios. A literature review contextualizes the anatomical features, surgical risks, and management strategies for this rare combination.

## 1 Introduction

Portal annular pancreas (PAP) is an exceedingly rare developmental anomaly resulting from the abnormal fusion of the ventral and dorsal pancreatic anlagen, leading to a ring of pancreatic tissue completely surrounding the portal vein (PV) or superior mesenteric vein (SMV). Its reported incidence ranges from 0.8% to 2.5% based on imaging and surgical series (1). However, its true prevalence may be underestimated because it is often missed or misdiagnosed as a locally advanced lesion in the pancreatic head or uncinate process. In a retrospective study involving patients undergoing pancreaticoduodenectomy (PD), the incidence of PAP was 0.4% (2). Concurrently, anatomical variations of the hepatic arteries are common, with a common hepatic artery (CHA) originating from the superior mesenteric artery (SMA) (Michels type IX) having an incidence of approximately 0.4%–4.5% (3, 4). While individually uncommon, the simultaneous presence of PAP and an aberrant CHA with a periampullary or distal bile duct tumor creates a formidable surgical landscape. The annular pancreas obscures the standard plane for pancreatic neck transection and increases postoperative pancreatic fistula (POPF) risk (5), while the aberrant artery, if unrecognized, risks catastrophic intraoperative injury and hepatic ischemia (6). This report details the successful management of such a case, emphasizing the diagnostic and technical considerations, and reviews the relevant literature.

## 2 Case presentation

A 55-year-old male presented with a one-month history of progressive, painless jaundice, accompanied by pruritus, dark urine, and clay-colored stools for the preceding ten days. He reported anorexia and an unintentional weight loss of approximately 5 kg. His past medical history included diabetes mellitus, status post percutaneous coronary intervention, and an appendectomy. Physical examination revealed marked scleral and cutaneous icterus. The abdomen was soft with mild tenderness in the right upper quadrant. The gallbladder was not palpable, and Murphy's sign was negative.

Laboratory tests confirmed obstructive jaundice: total bilirubin 249.1 µmol/L, direct bilirubin 139.7 µmol/L, with markedly elevated alkaline phosphatase (233 U/L) and gamma-glutamyl transferase (159 U/L). The tumor marker CA 19–9 was elevated at 44.3 U/mL. Multiphasic contrast-enhanced computed tomography (CT) (120 kVp, 250 mAs, slice thickness 1.25 mm; arterial and portal venous phase protocols with non-ionic iodinated contrast) demonstrated segmental wall thickening with enhancement in the mid-to-distal common bile duct (CBD), with proximal biliary dilation (15 mm in diameter) and periductal soft-tissue infiltration, suspicious for cholangiocarcinoma. Three-dimensional CT angiographic reconstruction was performed using multimodal medical image processing software (uIGS-M8 workstation, United Imaging Healthcare) with volume rendering and maximum intensity projection techniques, revealing two significant anatomical variants: (1) a complete portal annular pancreas (PAP) — pancreatic tissue encircling the superior mesenteric vein/portal vein (SMV/PV) confluence, extending approximately 12 mm posterior to the PV in an infrasplenic configuration; and (2) an aberrant common hepatic artery (CHA) originating from the superior mesenteric artery (SMA), coursing posterior to the pancreas, anterior to the PV, and lateral to the CBD, ascending to the hepatoduodenal ligament (Michels type IX), with the gastroduodenal artery (GDA) arising from the CHA at the superior border of the pancreas (Figure 1). Upper abdominal magnetic resonance (MR) further confirmed the anteportal course of the main pancreatic duct (MPD) (Figure 2), with no pancreas divisum, classifying the PAP as Joseph Type IIIb.

**Figure 1:**
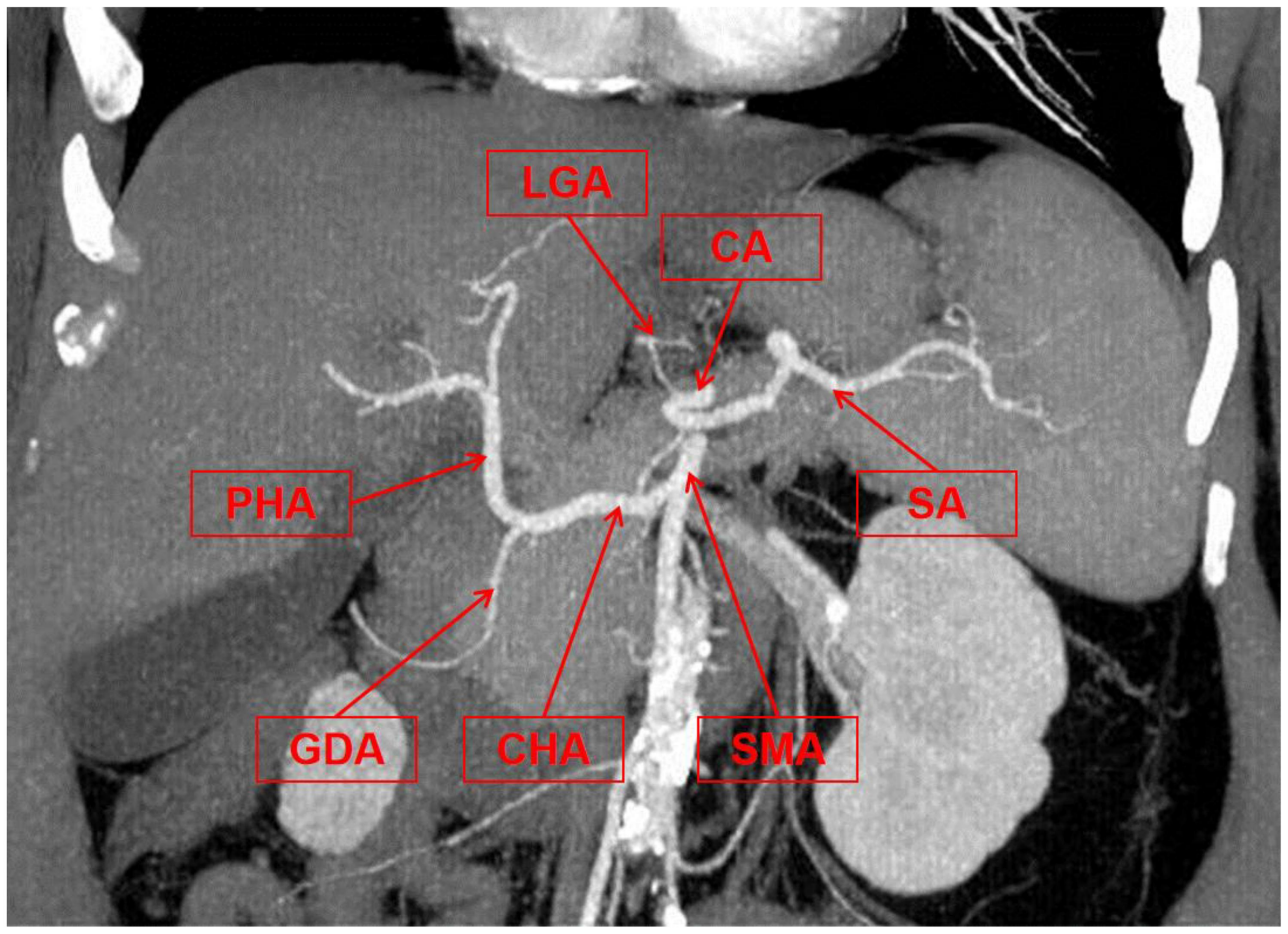
Three-dimensional contrast-enhanced CT angiographic reconstruction (coronal view) demonstrating the aberrant common hepatic artery (CHA) originating from the superior mesenteric artery (SMA), consistent with michels type IX classification. The vascular anatomy is labeled as follows: celiac axis (CA), left gastric artery (LGA), splenic artery (SA), proper hepatic artery (PHA), gastroduodenal artery (GDA), common hepatic artery (CHA), and superior mesenteric artery (SMA). Note the complete course of the CHA arising from the SMA, ascending posterior to the pancreatic parenchyma and anterior to the portal vein, with the GDA branching at the superior border of the pancreas. This aberrant CHA serves as the sole arterial inflow to the liver, underscoring the critical need for preoperative vascular mapping prior to pancreaticoduodenectomy.

**Figure 2:**
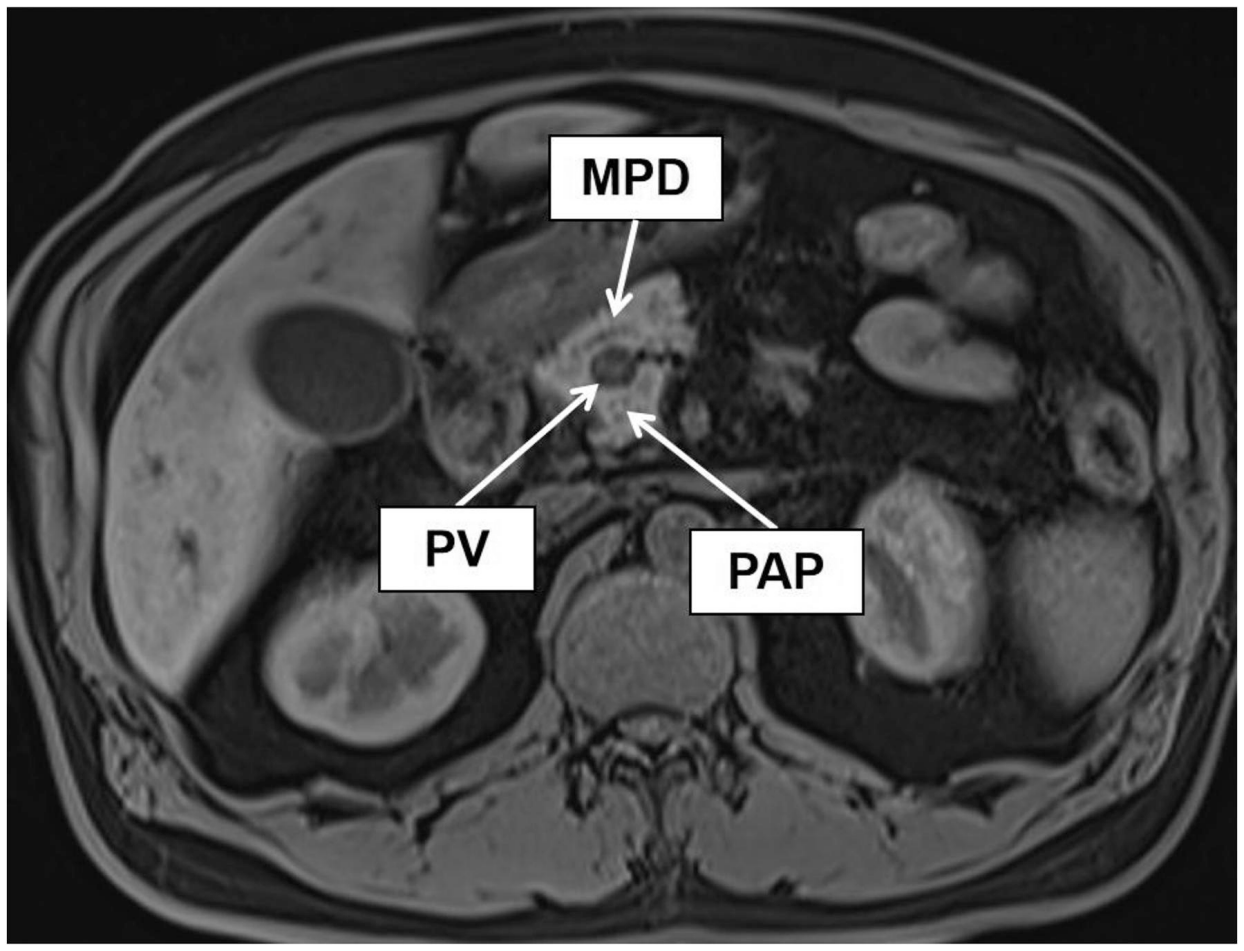
Axial MR image illustrating the portal annular pancreas (PAP) with the main pancreatic duct (MPD) coursing anterior to the portal vein (PV). The anteportal trajectory of the MPD and the absence of a dominant duct within the retroportal pancreatic tissue confirm the Joseph Type III configuration. This anatomical variant is decisive for surgical planning, as it permits safe transection of the retroportal pancreatic parenchyma without disrupting ductal continuity.

Preoperatively, percutaneous transhepatic biliary drainage (PTBD) was performed for severe obstructive jaundice. A classical pancreaticoduodenectomy (Whipple procedure) was undertaken, with intraoperative confirmation of a distal CBD tumor, complete PAP (Joseph Type IIIb), and aberrant CHA — all consistent with preoperative 3D imaging (Figure 3). The core challenge lay in simultaneously managing two rare variants. For the PAP, a phased two-stage transection was employed: the pancreas was first divided at the pancreatic neck, anterior to the PV, in the standard plane, identifying a single anteportal MPD (∼3 mm in diameter) with no accessory duct; the retroportal tissue was then meticulously dissected off the anterior and lateral walls of the PV and divided on the left side of the PV, allowing *en bloc* resection of the duct-less posterior tissue with the specimen to eliminate blind-stump fistula risk. For the aberrant CHA, the vessel was traced from its SMA origin to the proper hepatic artery; the GDA was ligated at the superior pancreatic border, and the CHA was preserved throughout its retropancreatic course as the sole hepatic arterial inflow (Figure 4). Frozen-section margins were negative (R0). Reconstruction comprised end-to-side pancreaticojejunostomy (modified Blumgart technique, duct-to-mucosa with a 5-Fr stent) at the anterior remnant surface containing the MPD, with the posterior surface oversewn using 4–0 polypropylene, followed by hepaticojejunostomy and gastrojejunostomy. Total operative time was 4 h 50 min; estimated blood loss was 300 mL with no vascular injuries.

**Figure 3:**
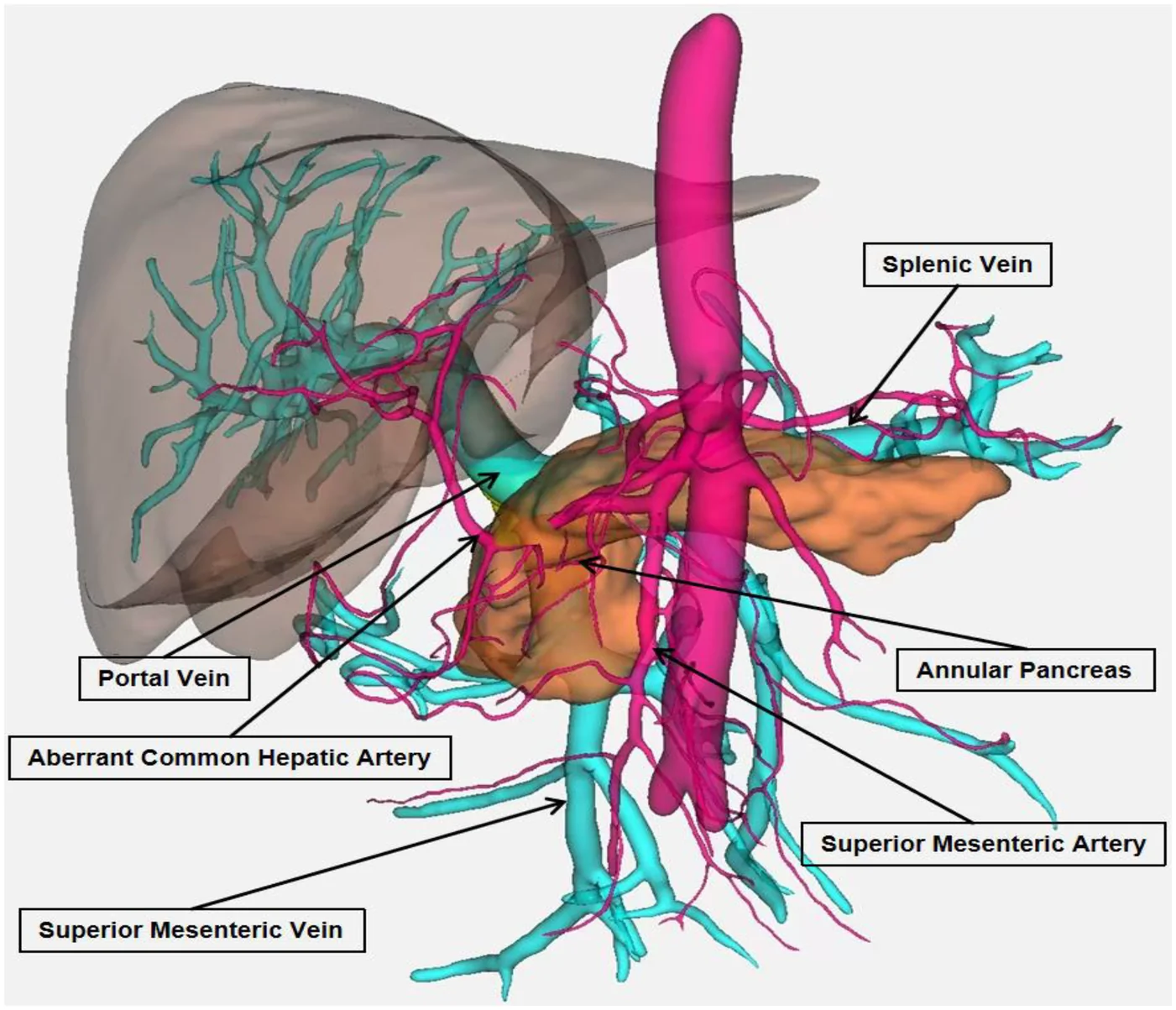
Three-dimensional vascular and parenchymal reconstruction depicting the complex spatial relationships among the annular pancreas, aberrant common hepatic artery, portal vein, splenic vein, superior mesenteric artery, and superior mesenteric vein. This reconstruction was generated using multimodal medical image processing software and provided essential preoperative guidance for surgical planning.

**Figure 4:**
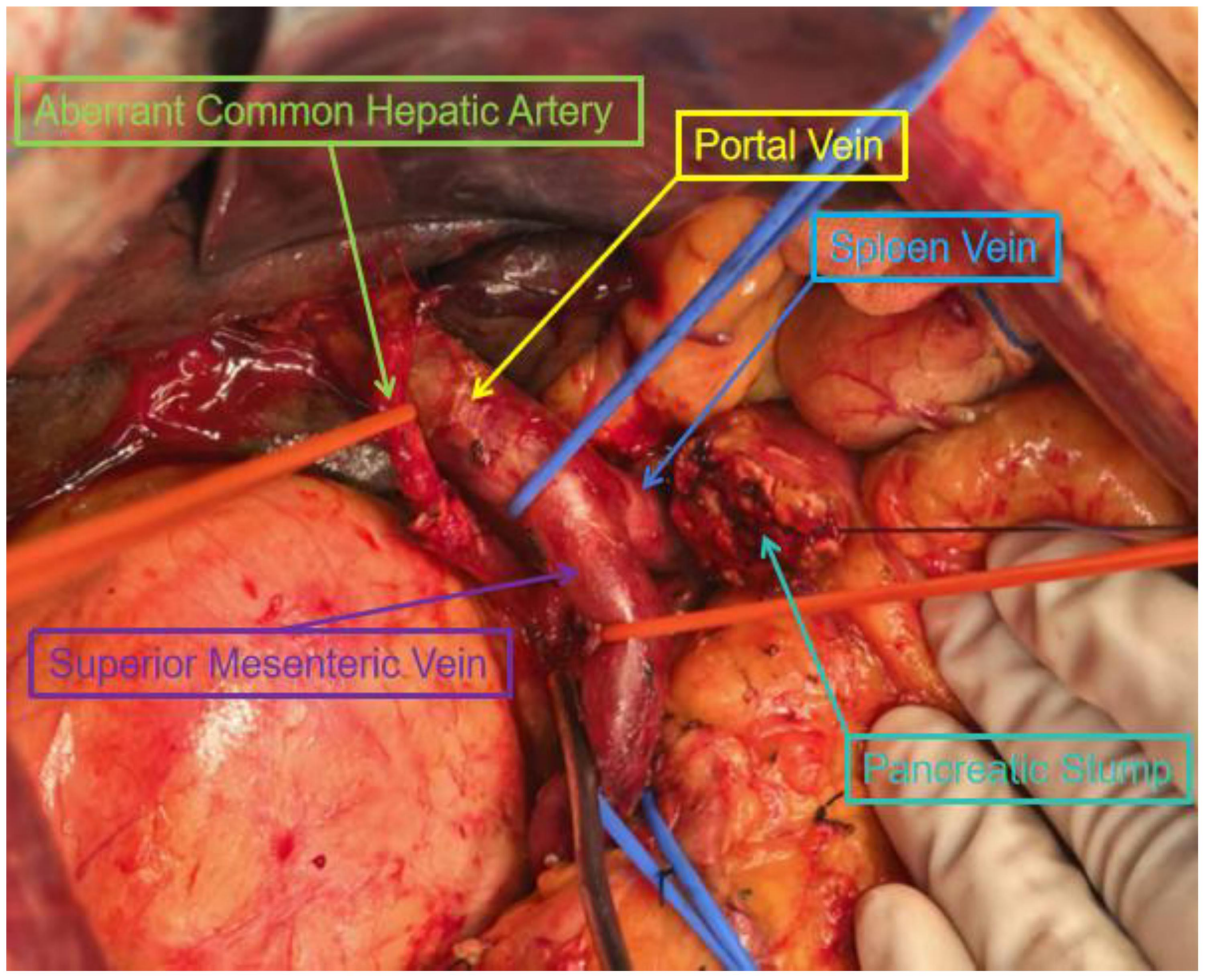
Intraoperative photograph taken after completion of pancreaticoduodenectomy, demonstrating the surgical field following the phased two-stage pancreatic transection. The aberrant common hepatic artery is preserved along its retropancreatic course as the sole hepatic arterial inflow. The portal vein, splenic vein, and superior mesenteric vein are skeletonized, and the pancreatic stump is shown.

Only a biochemical leak was observed postoperatively, which did not meet the diagnostic threshold for clinically relevant postoperative pancreatic fistula (POPF, ISGPS Grade B/C) per the 2016 International Study Group of Pancreatic Surgery (ISGPS) criteria (7). The patient did not develop delayed gastric emptying (DGE), post-pancreatectomy hemorrhage (PPH), or bile leakage during the perioperative period. Surgical drains were removed on postoperative day (POD) 6, and the patient was discharged on POD 15 following an uneventful recovery. Final pathology revealed a moderately to poorly differentiated adenocarcinoma of the distal common bile duct (1.7 × 1.2 cm), with a measured depth of invasion of 8 mm (AJCC 8th edition T2 category: 5–12 mm) and perineural invasion. Of 22 regional lymph nodes dissected, 4 were positive for metastatic carcinoma (pN2). All surgical margins were negative, achieving R0 resection. According to the American Joint Committee on Cancer (AJCC) 8th edition staging system, the final pathological stage was pT2N2M0, Stage IIIA (8).

## 3 Discussion

This case highlights the critical importance of individualized, anatomy-oriented surgical strategies guided by high-quality preoperative imaging in managing the combined challenges of PAP and an aberrant CHA during PD.

PAP is a rare congenital anomaly characterized by fusion of the pancreatic uncinate process with the pancreatic body, resulting in circumferential encasement of the PV or SMV. This anatomical variation not only increases the technical difficulty of pancreatic transection but also significantly elevates the risk of POPF due to the presence of a posterior pancreatic remnant (1).

To standardize its anatomical description and facilitate surgical planning, two principal classification systems have been proposed. In 2009, Karasaki et al. (9) classified PAP into three types based on the vertical relationship between the uncinate–body fusion site and the splenic vein–portal vein (SV–PV) confluence: Type A (suprasplenic, fusion located above the SV–PV confluence), Type B (infrasplenic, fusion located below the confluence), and Type C (mixed, fusion involving both above and below the confluence). This system provides a fundamental morphological framework for understanding the vertical distribution of the annular tissue.

In 2010, Joseph et al. (10) established a classification scheme based on the topographic relationship between the MPD and PV, which bears greater relevance to surgical planning and risk stratification. Within this scheme, Type I represents fusion of the ventral bud with the pancreatic body with a retroportal MPD; Type II denotes Type I complicated by pancreas divisum; Type III is characterized by isolated uncinate–body fusion with an anteportal MPD. Integrating this duct-based classification with the morphological stratification described by Karasaki et al., each Joseph type can be further subclassified into a (suprasplenic), b (infrasplenic), and c (mixed) variants, forming nine composite subtypes ranging from Ia to IIIc (9, 11). The systematic review published by Harnoss et al. (11) further summarized the clinical value of these dual classification systems and confirmed that PAP confers an increased risk of POPF.

In the present case, the patient was classified as Joseph Type IIIb, characterized by the MPD coursing anterior to the PV (Type III) with encircling pancreatic tissue located below the SV–PV confluence (subtype b, infrasplenic). The basis for this classification was established through combined CT and MR imaging: (1) The MPD was clearly visualized on both portal venous phase CT and MR as coursing through the anteportal pancreatic tissue, anterior to the PV; (2) the absence of a dominant duct in the retroportal pancreatic segment was determined by demonstrating on serial CT slices and MR source images that the MPD maintained continuity within the anteportal pancreatic tissue from the pancreatic body to the duodenal side, with no ductal structure identified in the tissue posterior to the PV on any imaging sequence; and (3) the encircling pancreatic tissue was located below the level of the SV–PV confluence, consistent with the infrasplenic (subtype b) configuration.

This configuration has important surgical implications. The anterior position of the MPD increases the risk of inadvertent ductal injury during standard pancreatic transection, while the infraconfluence encasement necessitates meticulous dissection along the distal PV. Moreover, the presence of a posterior pancreatic segment lacking a dominant duct predisposes to the formation of a blind pancreatic stump, which is a well-recognized risk factor for POPF (1). In Type III PAP, the retroportal tissue does not contain the dominant MPD, allowing for its *en bloc* resection with the specimen without sacrificing ductal continuity—a principle that directly guided our surgical approach.

The phased pancreatic transection technique employed in this case warrants detailed discussion. In PAP, standard single-plane transection at the pancreatic neck leaves a posterior remnant encircling the PV, which may contain ductal tissue and predispose to POPF. Our two-stage approach created two transection surfaces on the remnant: the anterior surface (containing the MPD) for pancreaticojejunostomy and the posterior surface (without a dominant duct), which was oversewn. The posterior pancreatic tissue was resected *en bloc* with the specimen, eliminating a potential source of fistula from a blind, duct-less posterior stump (12). This strategy is supported by the systematic review by Modak et al. (2), which reported that *en bloc* extended resection of the retroportal tissue was associated with numerically lower clinically relevant POPF rates (22.7%) compared to suturing (37.5%) or stapling (53.9%) of the posterior stump.

The coexistence of an aberrant CHA arising from the SMA, corresponding to Michels type IX (3), further increases operative complexity. In this variant, the CHA represents the sole arterial inflow to the liver; therefore, misidentification as the GDA or inadvertent injury during dissection can result in catastrophic hepatic ischemia (13). Preoperative three-dimensional vascular reconstruction is essential for accurate anatomical delineation of the complete arterial course, including the GDA origin and the relationship of the CHA to the pancreas, PV, and CBD. Intraoperatively, meticulous dissection of the hepatoduodenal ligament, early identification of the GDA origin from the aberrant CHA, and strict preservation of the CHA—particularly at its origin from the SMA—are critical to ensure hepatic perfusion (14).

The concurrence of these anatomical variations in the setting of malignant distal biliary obstruction creates a “perfect storm” of surgical complexity. Although the role of routine preoperative biliary drainage (PBD) in PD remains debated, a recent meta-analysis by Zhang et al. (15) demonstrated that preoperative biliary drainage increases the risk of postoperative complications without clear overall benefits, but may be considered for high-risk patients such as those with severe infections or progressive jaundice. In our case, PTBD was selectively employed given the patient's severe obstructive jaundice (total bilirubin 249.1 µmol/L) and the anticipated prolonged and technically demanding procedure, which is consistent with the selective use of PBD recommended for high-risk patients (15).

Postoperatively, computed tomography serves as the primary imaging modality for the evaluation of early complications following pancreaticoduodenectomy, including fluid collections, abscess formation, pancreatic fistula, hemorrhage, and vascular complications (16). In our case, postoperative recovery was uneventful, and no imaging-detected complications were observed.

In summary, this case underscores that successful management of complex pancreatic surgery relies on a thorough understanding of anatomical variations, precise preoperative planning, and tailored intraoperative strategies. The adoption of a phased pancreatic transection technique combined with vigilant vascular preservation can effectively mitigate operative risks and contribute to favorable surgical outcomes.

## Data Availability

The original contributions presented in the study are included in the article/Supplementary Material, further inquiries can be directed to the corresponding author.
